# Genomic sequencing combined with marker-assisted breeding effectively eliminates potential linkage drag of a target gene: a case study in tobacco

**DOI:** 10.3389/fpls.2025.1666106

**Published:** 2025-09-24

**Authors:** Wenxia Gao, Wen Yu, Jinbin Lin, Zhenfu Zhao, Ningning Yin, Juxian Lai, Yazhi Cheng, Likun Huang, Chunying Li, Shunhui Chen, Weiren Wu, Shengxin Wu

**Affiliations:** ^1^ Key Laboratory of Genetics, Breeding and Multiple Utilization of Crops, Ministry of Education, College of Agriculture, Fujian Agriculture and Forestry University, Fuzhou, China; ^2^ Fujian Key Laboratory of Crop Breeding by Design, College of Agriculture, Fujian Agriculture and Forestry University, Fuzhou, China; ^3^ Key Laboratory of Biological Breeding for Fujian and Taiwan Crops, Ministry of Agriculture and Rural Affairs, College of Agriculture, Fujian Agriculture and Forestry University, Fuzhou, China; ^4^ Institute of Tobacco Science, Fujian Tobacco Monopoly Administration, Fuzhou, China

**Keywords:** linkage drag, genomic sequencing, marker-assisted breeding, tobacco mosaic virus (TMV), N gene, disease resistance

## Abstract

Linkage drag frequently impedes the utilization of beneficial genes from wild species in crop improvement. The *N* gene from *Nicotiana glutinosa* confers strong resistance to tobacco mosaic virus (TMV) but introduces linkage drag when introgressed into cultivated tobacco (*Nicotiana tabacum*). To address this issue, we sequenced the TMV-resistant flue-cured tobacco line 0970A and carried out comparative genomic analysis. Additionally, we used molecular markers to screen a BC_4_F_1_ population derived from the cross between 0970A and an elite flue-cured tobacco variety CB-1 (recurrent parent). As a result of sequencing 0970A, the *N* gene was located at the end of chromosome Nt11. The comparative genomic analysis showed that 0970A inherited approximately 3.74 Mb of *N. glutinosa* DNA (*N*-fragment) from its donor, Coker 176. From screening the BC_4_F_1_ population with molecular markers, a recombinant was identified. This recombinant had a significantly reduced *N*-fragment (~270 kb), which minimized the linkage drag while still maintaining resistance to TMV. This research indicates that the combination of genome sequencing and marker-assisted breeding can be successfully applied to reduce linkage drag. The findings offer valuable resources for breeding tobacco with resistance to TMV.

## 1 Introduction

Introducing favorable genes from wild species into cultivated varieties via sexual hybridization is a prevalent practice in plant breeding. Nevertheless, when transferring the target beneficial genes, some linked unfavorable genes may also be introduced, a phenomenon referred to as linkage drag. Hence, eliminating linkage drag (that is, breaking the linkage between favorable and unfavorable genes) stands as a crucial task in leveraging wild germplasm resources for plant breeding (Huang et al., 2022). However, given that it remains unclear which of the linked genes are unfavorable, all the linked genes from wild species are regarded as potentially harmful. Therefore, an effective strategy to alleviate linkage drag is to minimize the genomic segments from wild species that are co-introduced with the target gene. To achieve this, it is necessary to accurately identify the alien fragment introgressed along with the target gene from the wild-species parent (Huang et al., 2022).

Tobacco mosaic virus (TMV) is a single-strand sense RNA virus that causes a common disease in cultivated tobacco (*Nicotiana tabacum*) known as tobacco mosaic disease, resulting in significant economic losses in tobacco production (Creager, 2022). Developing TMV-resistant tobacco varieties is the most cost-effective and efficient method to manage tobacco mosaic disease. The dominant *N* gene from *Nicotiana glutinosa* (Holmes, 1938) is the most commonly used TMV-resistant gene in tobacco breeding due to its strong resistance, simple inheritance, and easy identification, although there are some other genes discovered for TMV resistance in *N. rustica* sp (Holmes, 1937; Nifong, 2008) and *N. repanda* (Burk, 1967; Gwynn, 1986; Bates, 1990).

The *N* gene confers resistance to TMV in plants by initiating a hypersensitivity response, preventing the spread of the virus (Marathe et al., 2002). In 1914, Allard first observed necrotic lesions on the leaves of *N. glutinosa* infected with TMV. Later, through classical genetic experiments, Holmes (1938) demonstrated that this necrotic response was controlled by a dominant gene, which he named *N*. Whitham et al. (1994) successfully cloned the *N* gene by utilizing the insertion mutation caused by the maize Ac transposon. The N protein consists of three domains. The first domain is the N-terminal TIR domain, which shares similarities with the cytoplasmic domain of Toll and interleukin-1 receptor (IL-1R) in drosophila and humans. The second domain is the nucleotide-binding (NB) domain located in the middle. The third domain is the C-terminal leucine-rich repeat (LRR) domain. Therefore, the *N* gene falls under the *R* gene classification of the TIR-NB-LRR class (Baker et al., 1997; Marathe et al., 2002).

Different researchers have introduced the *N* gene into *N. tabacum* through interspecific hybridization and multi-generation backcross, as documented in several studies (Holmes, 1938; Gerstel, 1943; Mallah, 1943; Gerstel, 1945a, 1946). This genetic modification led to the development and widespread cultivation of TMV-resistant burley tobacco varieties (Chaplin and Mann, 1978; Legg et al., 1979; Lewis et al., 2005). However, the TMV-resistant flue-cured tobacco varieties obtained through the same gene have exhibited reduced yield and quality, failing to gain popularity among farmers. Research indicates that the negative impact of introducing the *N* gene is attributed to linkage drag rather than the gene’s inherent pleiotropic nature (Chaplin et al., 1961, 1966; Chaplin and Mann, 1978; Legg et al., 1979; Lewis et al., 2007).

With the cloning of the *N* gene, researchers have explored introducing it into tobacco using transgenic technology to circumvent linkage drag issues (Lewis et al., 2007). Nonetheless, the limited commercial use of transgenic tobacco necessitates reducing or eliminating *N* gene linkage drag through backcrossing and molecular marker-assisted selection. To effectively address the linkage drag associated with the *N* gene, understanding the location and size of the foreign fragment carrying the *N* gene (referred to as *N*-fragment here for convenience) from *N. glutinosa* in the *N. tabacum* genome is essential.

The *N*-fragment introduced into *N. tabacum* by Holmes (1938) was found to be integrated on chromosome H (Gerstel, 1945b), but the *N*-fragments introduced into *N. tabacum* by other researchers might be integrated on different chromosomes (Lewis et al., 2005). The *N. tabacum* genome has been sequenced (Sierro et al., 2014; Edwards et al., 2017; Sierro et al., 2024; Wang et al., 2024). However, it is unclear which chromosome corresponds to the chromosome H. In addition, the specific integration position of the *N*-fragment on the chromosome also remains uncertain.

Several *N. tabacum* varieties or lines that are resistant to TMV have been developed from the original *N*-gene introgressed lines. Lewis et al. (2005) utilized AFLP markers to examine 15 TMV-resistant *N. tabacum* materials and found significant variation in the size of *N*-fragments among them. Among these materials, Coker 176 and Burley 21 appear to have the smallest *N*-fragment. Therefore, these two varieties are the optimal candidates as an *N* gene donor parent for breeding TMV-resistant tobacco. However, the study by Lewis et al. (2005) was unable to determine the precise size of the *N*-fragment in each material.

Line 0970A is a flue-cured tobacco inbred line resistant to TMV. It was developed by the Institute of Tobacco Science, Fujian Tobacco Monopoly Administration, China, with Coker 176 serving as the source of the *N* gene. This line functions as a donor parent for TMV resistance in flue-cured tobacco breeding. Cuibi-1 (abbreviated as CB-1) is a major elite cultivated variety of flue-cured tobacco in Fujian province, China. However, it is highly susceptible to TMV. To enhance its resistance, introducing the *N* gene into CB-1 has become a key objective in its genetic improvement. For this purpose, we used 0970A as the donor parent and CB-1 as the recurrent parent, and incorporated the *N* gene through successive backcrossing. Nevertheless, as with other TMV resistance breeding programs in flue-cured tobacco, this effort also faces the challenge of minimizing the undesirable linkage drag associated with the *N* gene.

In this study, through genome sequencing and marker-assisted breeding techniques, we accurately determined the location and size of the *N*-fragment within the 0970A genome, along with the genes it encompasses, and significantly minimized the potential linkage drag when introducing the *N* gene from 0970A into the genetic background of CB-1. Our study successfully demonstrated the efficacy of this approach, presenting a compelling example of the viability of integrating genome sequencing and marker-assisted breeding to reduce or even completely eliminate linkage drag. The data and TMV-resistant lines acquired in this study will facilitate the efficient utilization of the *N* gene in TMV-resistance breeding programs and assist in circumventing linkage drag issues in tobacco cultivation.

## 2 Materials and methods

### 2.1 Plant materials

Four plant materials were utilized in this study, including *N. glutinosa* (the primary source of *N* gene), Coker 176 (a flue-cured tobacco variety possessing the *N* gene), 0970A (a flue-cured tobacco inbred line possessing the *N* gene inherited from Coker 176), and CB-1 (a TMV-susceptible elite flue-cured tobacco variety, and one of the parents of 0970A).

### 2.2 *In vitro* identification for TMV resistance

To enhance the efficiency of TMV resistance identification and to avoid the impact of TMV inoculation on the growth of tobacco plants, we employed an *in vitro* identification method (Yin et al., 2023) in this study. Diseased tobacco leaves were collected, ground, and filtered to prepare a TMV solution. At the seven-leaf stage of the tobacco seedlings, one well-grown leaf from each plant was selected. A toothbrush was used to apply the TMV solution to the surface of the leaf, which was then placed in a self-sealing bag and incubated in a growth chamber at 26 °C with a 12-hour light and 12-hour dark cycle. After 72 hours, the leaf conditions were observed. Leaves containing the *N* gene (resistant to TMV) would form immune spots, while those without the *N* gene (susceptible to TMV) would not form such spots (Yin et al., 2023).

### 2.3 Sequencing and assembly of 0970A genome

The genome of 0970A was sequenced and assembled by Berry Genomics (Beijing). Genomic DNA was extracted from approximately 300 mg of fresh leaf tissue using the CTAB method. DNA quality was assessed using a NanoDrop™ ND-2000 spectrophotometer (Thermo Fisher Scientific), a Qubit™ fluorometer (Invitrogen, Q33238), and the CHEF Mapper™ XA Chiller System (Bio-Rad, 1703672). A high-fidelity (HiFi) library was prepared with the SMRTbell^®^ Prep Kit 3.0 (Pacific Biosciences, 102-182-700) and sequenced on the PacBio Revio^®^ system (Pacific Biosciences, 102-090-600). The raw reads were processed with SMRT software (Ardui et al., 2018) to generate HiFi reads, which were then assembled using Hifiasm (Cheng et al., 2021). The resulting contigs were subsequently deduplicated using Purge dups (Guan et al., 2020). Contig quality and assembly completeness were assessed with Quast (Mikheenko et al., 2018) and BUSCO (Manni et al., 2021). All software was run using default parameters.

### 2.4 Identification, location, and annotation of *N*-contig

The *N* gene sequence (gene ID LOC107824161) was obtained from the public database GenBank. This sequence was then compared with all the contigs of 0970A through the BLAST comparison tool to identify the contig containing the *N* gene (termed *N*-contig). The collinearity between the *N*-contig and the CB-1 genome (unpublished) was analyzed to identify the location of the *N*-contig in the tobacco genome using Minimap2 software under default parameter -x asm20 (Li, 2018). Annotation of the *N*-contig was achieved by annotating all contigs. Initially, repetitive sequence elements within all contigs were identified using RepeatModeler v2.0.5 software (Flynn et al., 2020) and subsequently masked using RepeatMasker v4.1.2.p1–1 software (Tempel, 2012). Three strategies were then employed to predict protein-coding genes. The first strategy involved transcriptome-based prediction, utilizing the tobacco transcriptomes (NCBI accession numbers: SRR1199073, SRR1199122, SRR1199197, SRR1199202) and hisat2 v2.2.1–6 software (https://daehwankimlab.github.io/hisat2). The second strategy was homology-based prediction, leveraging the annotation sets from tomato (GCF_036512215.1), tobacco TN90 (GCA_000715135.1), and chili pepper (GCA_002878395.3). The third strategy was *de novo* prediction, employing GeneMark-ETP v1 software (Brüna et al., 2020) and AUGUSTUS v3.3.3 (Stanke et al., 2004). These results were integrated to generate an initial annotation file using Braker3 v3.0.8–0 software (Gabriel et al., 2024) and further refined using TSEBRA v1.2.5 software (Gabriel et al., 2021), resulting in the final protein annotation file.

### 2.5 Homology analysis of *N*-contig

The genomic DNA of CB-1, Coker 176, and *N. glutinosa* was extracted using the Plant Genomic DNA Kit (TIANGEN, Beijing) and the three DNA samples were subsequently sent to Berry Genomics (Beijing) for deep sequencing (at least 80 Gb each in depth) on the DNBSEQ-T7 platform. For each sample, adapter-trimmed reads were aligned to all contigs of 0970A using BWA-MEM2 v2.2.1 (Vasimuddin et al., 2019) under default parameters, with the exception of the -k option (minimum seed length), which was increased from 19 to 30 to enhance mapping stringency. The average depth of reads mapped to the *N*-contig was calculated every 10 kb using Samtools v1.19 (Li et al., 2009).

### 2.6 Creation of recombinant lines with reduced linkage drag of the *N* gene

**Figure 1:**
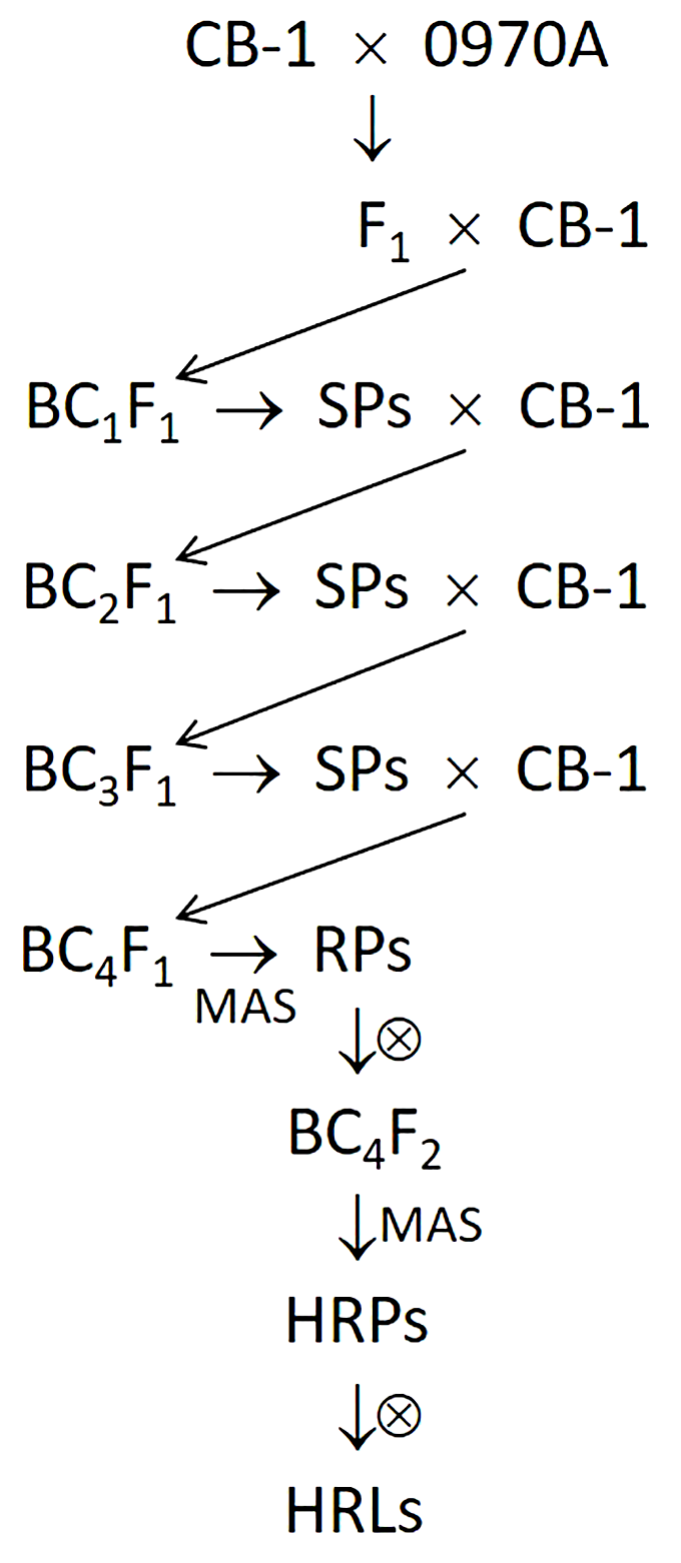
Procedure of introducing the *N* gene from 0970A into CB-1 with reduced linkage drag. SPs, selected plants resistant to TMV and most similar to the recurrent parent CB-1 in morphology. The selection was performed according to the phenotypes of the plants. MAS, marker-assisted selection. RPs, recombinant plants with heterozygous genotype at the *N* locus (*Nn*). HRPs, recombinant plants with homozygous genotype (*NN*). HRLs, recombinant lines with homozygous genotype (*NN*).

To introduce the *N* gene from 0970A into CB-1 and disrupt and eliminate the linkage drag associated with the *N* gene, we crossed 0970A with CB-1 and performed consecutive backcrosses up to the BC_4_F_1_ generation. In each generation, we selected a small number of plants that were morphologically most similar to CB-1 but retained the *N* gene and backcrossed them with CB-1. In the BC_4_F_1_ generation, we established a large population. We then employed a molecular marker (P371) developed based on the sequencing data to screen TMV-resistant plants for chromosomal recombination events within the target region (the linkage drag of the *N* gene from *N. glutinosa*). Such recombination events would lead to the partial elimination of the linkage drag. For clarity, we designated TMV-resistant plants containing the target recombination events as “recombinant plants,” while those without such events were termed “non-recombinant plants.” The identified recombinant plants were self-pollinated to produce the corresponding BC_4_F_2_ lines. Within these BC_4_F_2_ lines, we used a linked molecular marker (NR5) developed based on the sequencing data to identify plants with a homozygous *N*-gene genotype. These plants were further self-pollinated to obtain the corresponding *N*-gene homozygous lines, which are the recombinant lines with reduced linkage drag wanted. The above procedure is illustrated in **Figure 1**.

### 2.7 Molecular analysis of recombinant lines

To precisely determine the locations of recombination events occurred within the target region, the “homology analysis of *N*-contig” approach described earlier was applied to the identified recombinant lines. Specifically, each recombinant line was subjected to deep sequencing (approximately 40 Gb in depth), and the resulting reads were mapped to all contigs of 0970A. In addition, a non-recombinant line was analyzed in the same manner for comparative purposes.

## 3 Results

### 3.1 Sequencing and assembly of 0970A genome

A total of 6,892,630 HiFi reads were obtained from sequencing the genome of 0970A, with an average read length of 17.3 kb, a read N50 of 17,254 bp, and an average sequencing depth of 29 times the genome. These HiFi reads were then assembled and redundancies were eliminated, resulting in 554 contigs. Among these contigs, the N_50_ length was 81,799,233 bp, and the N_75_ length was 53,933,392 bp. Notably, approximately 80 contigs comprehensively covered the entire genome. The assessment using BUSCO revealed that the assembled genome sequence exhibited an impressive 99% completeness, underscoring the exceptional quality of the sequencing and assembly process.

### 3.2 Identification, annotation, and chromosomal location of *N*-contig

**Figure 2:**
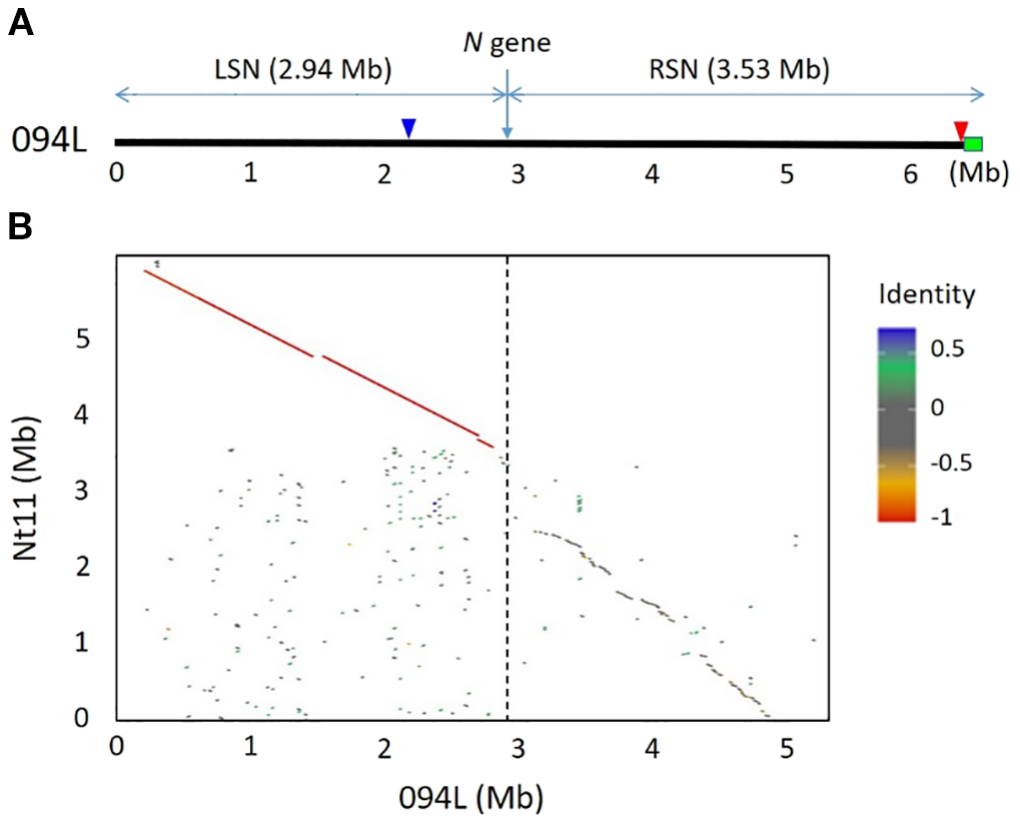
Contig 094L and its collinearity with the Nt11 chromosome terminus of CB-1. **(A)** Schematic diagram of 094L showing the position of the *N* gene as well as the LSN and RSN regions. The green rectangular represents telomere. The red and blue arrowheads indicate the positions of markers P371 and NR5, respectively. **(B)** Collinearity comparison between 094L and the Nt11 terminus of CB-1. The identity scale is on the right. The red color indicates complete identity but in opposite direction. The vertical broken line indicates the position of the *N* gene.

Upon aligning the *N* gene sequence with all contigs of 0970A, it was determined that the *N* gene resided on contig PTG000094L, hereafter referred to as 094L (the detailed information of 094L, including full sequence and predicted genes, can be found at https://ngdc.cncb.ac.cn/genbase/search/gb/C_AA098042). The length of the *N* gene sequence used as the query was 7,345 bp, exhibiting a perfect match with the target sequence (E-value = 0, Score = 13,564). Notably, one end of contig 094L was identified as a telomere sequence with a repeat unit of TTTAGGG, suggesting that the contig represented a chromosomal terminus. This contig spanned 6.47 Mb in length, with the *N* gene located at 2.94 Mb from the non-telomere end. For convenience, we hereafter call the sequence of the contig 094L from the non-telomere end to the *N* gene and that from the telomere end to the *N* gene as the left side of *N* gene (LSN) and right side of *N* gene (RSN), respectively (**Figure 2A**).

A total of 167 genes were predicted on contig 094L, with 62 on the LSN and 104 on the RSN (**Supplementary Table S1**). Interestingly, within the LSN, three genes were also annotated to encode the TMV resistance protein N, namely, *ptg94lG0000310.t1* (abbreviated as *G31*) located at 1.94 Mb, *ptg94lG0000390.t1* (*G39*) at 2.26 Mb, and ptg94lG0000440.t1 (*G44*) at 2.43 Mb (**Supplementary Table S1**). However, they all were shorter than the full-length of the *N* gene sequence, measuring 6,419 bp (*G31*), 4,732 bp (*G39*), and 7,279 bp (*G44*), respectively, suggesting that they are only homologs of the *N* gene.

Since 094L was identified as the terminal sequence of a chromosome, we tried to locate the *N* gene on the chromosome by conducting collinearity analysis between 094L and the terminal sequences of all chromosomes of CB-1. The results revealed a significant collinearity between a segment of 094L and chromosome 11, referred to as Nt11 in accordance with the established chromosome numbering system of the K326 genome sequence (**Figure 2B**), suggesting that the *N* gene must be located on this chromosome. It is important to highlight that the collinearity between 094L and the corresponding CB-1 genome sequence is not consistent throughout. Specifically, the LSN exhibited a good collinearity with the CB-1 sequence, whereas the RSN displayed no collinearity with the CB-1 sequence (**Figure 2B**), implying that the RSN might not originate from *N. tabacum*.

### 3.3 Homology analysis of the sequences flanking the *N* gene

**Figure 3:**
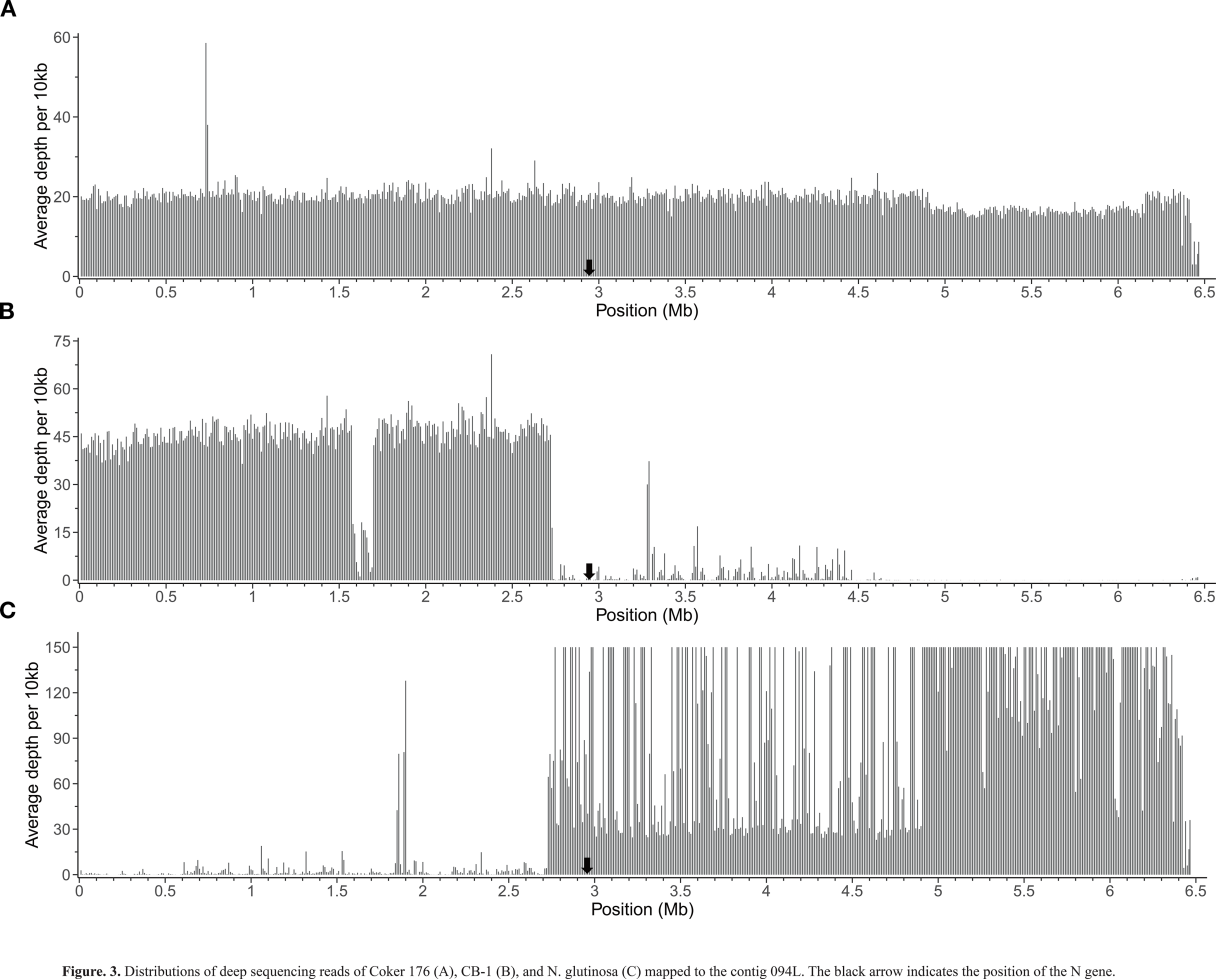
Distributions of deep sequencing reads of Coker 176 **(A)**, CB-1 **(B)**, and *N. glutinosa* **(C)** mapped to the contig 094L. The black arrow indicates the position of the *N* gene.

To precisely determine the homology of the sequences flanking the *N* gene, we performed deep sequencing of the genomes of CB-1, Coker 176, and *N. glutinosa*. We then mapped their reads to all contigs of 0970A and analyzed the mapping results for 094L (the reads of CB-1, Coker 176, and *N. glutinosa* mapped to 094L can be found at https://ngdc.cncb.ac.cn/bioproject/browse/PRJCA030530). The reads from Coker 176 uniformly covered the entire 094L with an average depth of approximately 20 (**Figure 3A**), indicating that the whole 094L is homologous to Coker 176. This finding aligns with the fact that Coker 176 is the donor parent of the *N* gene in 0970A. In contrast, the reads from CB-1 were predominantly mapped to the LSN and sparsely to the RSN (**Figure 3B**), suggesting that the LSN is homologous to CB-1. This result is consistent with the collinearity analysis (**Figure 2B**). Notably, the reads from CB-1 were evenly distributed across the LSN with an average depth of about 45, except for a small region (spanning from 1.55 Mb to 1.70 Mb) where the depth was significantly lower (**Figure 3B**). This indicates a structural variation between CB-1 and 0970A in that region, which the collinearity analysis suggests is an insertion in 0970A (**Figure 2B**). Conversely, the reads from *N. glutinosa* were primarily mapped to the RSN and barely to the LSN (**Figure 3C**), suggesting that the RSN is homologous to *N. glutinosa*. It is noteworthy that the depth distribution of *N. glutinosa* reads on the RSN was highly uneven. Although there was an overall depth plateau at around 30, reflecting the average sequencing depth of the *N. glutinosa* genome, there were many locations with extremely high depths (over 150), especially in the region beyond 4.92 Mb, where these high depths appeared almost continuously. The extremely high depths likely resulted from the presence of multiple copies of sequences dispersed throughout the *N. glutinosa* genome. Since these reads of multiple copies could not be mapped to other contigs of 0970A, they were exclusively mapped to the RSN, leading to the observed high depths.

### 3.4 Obtainment of a recombinant plant and its progeny homozygous lines

The results presented above demonstrate that the linkage drag associated with the *N* gene from *N. glutinosa* in 0970A (as well as in Coker 176) is mainly restricted to the RSN of contig 094L. To facilitate removing the associated linkage drag while introducing the *N* gene from 0970A into CB-1, we developed a PCR-based molecular marker, designated as P371, located approximately 3.46 Mb from the *N* gene at the RSN end (close to the telomere) of contig 094L (**Figure 2A**). The P371 marker (forward primer: 5’-CTAAAAACGGGTGAAGGGGT-3’; reverse primer: 5’-ACATTCGGAGGTCAGGTCAAT-3’) is a dominant marker that produces a specific 162 bp amplification product in the donor parent 0970A but not in the recipient parent CB-1. Using the *in vitro* identification method, we identified 4,762 TMV-resistant plants (carrying the *N* gene) from a population of 10,013 BC_4_F_1_ plants. All resistant plants were subsequently genotyped using the P371 marker, and one plant was found to lack the specific amplification product (**Supplementary Figure S1**). This finding indicates a recombination event between the P371 marker and the *N* gene, suggesting that a portion of the linkage drag, including the telomere, has been replaced with the corresponding segment from CB-1 in this plant. Therefore, this is a desired recombinant plant.

To identify individuals with a homozygous genotype for the *N* gene, we developed an SSR marker, designated as NR5, located approximately 740 kb from the *N* gene on the LSN of contig 094L (**Figure 2A**). The NR5 marker (forward primer: 5’-GGTCTATGTGCACCCTACCCT-3’; reverse primer: 5’-TTGGTTACGGCACGTAATGTT-3’) produces a 200 bp amplification product in 0970A and a shorter amplification product in CB-1 (**Supplementary Figure S2**). Since NR5 is closely linked to the *N* gene, individuals exhibiting only the 0970A-specific band are likely to be homozygous for the *N* gene. Using the NR5 marker, we screened 30 progeny plants derived from self-pollination of the recombinant plant and identified 8 plants with a homozygous genotype for the *N* gene. These 8 plants were further confirmed to carry the *N* gene using the *N* gene-specific marker N1+N2 (forward primer N1: 5’-CGTCGACACATTATGCCATC-3’; reverse primer N2: 5’-GAGGGGTCTTACCCCATTGT-3’) (Lewis et al., 2005). Self-pollination of these plants yielded 8 recombinant lines with a homozygous genotype for the *N* gene. Using a similar approach, we also established 3 non-recombinant control lines (homozygous for the *N* gene) from a single non-recombinant plant.

### 3.5 Molecular characterization of recombinant lines

**Figure 4:**
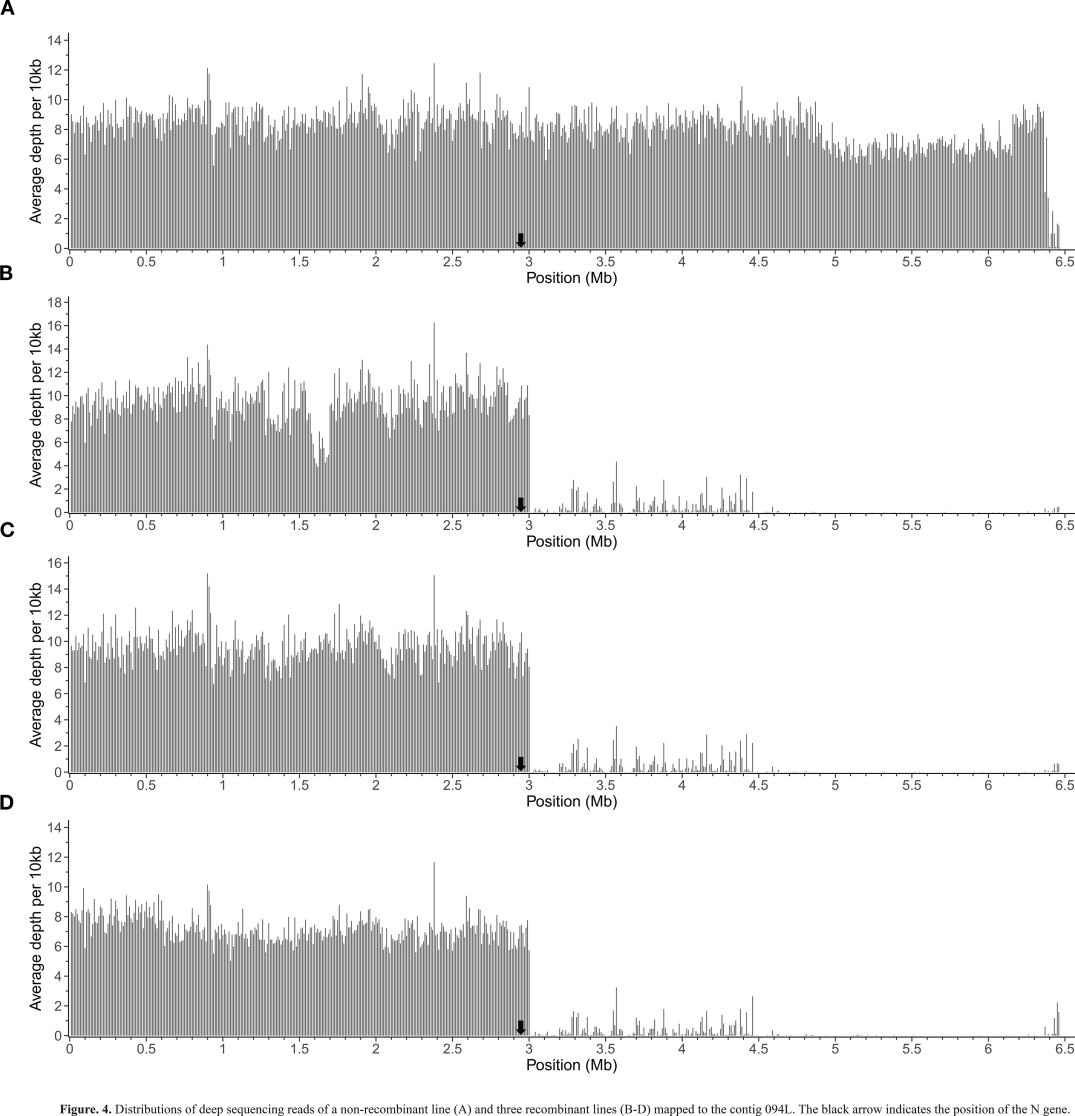
Distributions of deep sequencing reads of a non-recombinant line **(A)** and three recombinant lines **(B-D)** mapped to the contig 094L. The black arrow indicates the position of the *N* gene.

To precisely characterize the recombination events in the target region of the recombinant lines, we conducted deep sequencing analysis on 3 recombinant lines and 1 non-recombinant line (the reads of these lines mapped to 094L can be found at https://ngdc.cncb.ac.cn/bioproject/browse/PRJCA030530). As expected, the non-recombinant line retained the complete 094L sequence (**Figure 4A**), whereas the 3 recombinant lines exhibited extensive replacement of the RSN sequence, with the crossover point located approximately at the position of 3 Mb, only ~60 kb away from the *N* gene (**Figures 4B-D**). This finding indicates that the residual linkage drag associated with the *N* gene in the recombinant lines is minimal.

Furthermore, based on SNP markers revealed by deep sequencing, we found that the 3 recombinant lines contained 97.85%-98.72% of the genetic components from the recipient parent CB-1 (**Supplementary Table S2**), indicating a high degree of genetic background restoration. However, the non-recombinant line had a comparatively lower restoration rate of the CB-1 genetic background, containing only 93.42% of the genetic components from CB-1 (**Supplementary Table S2**).

## 4 Discussion

This study demonstrates that the *N* gene, introgressed from *N. glutinosa* into *N. tabacum*, is localized to the distal region of chromosome Nt11 in the *N. tabacum* genome, approximately 3.53 Mb from the telomere (**Figure 2A**; **Supplementary Table S1**). Homology analysis confirmed that 0970A has fully retained the *N* gene and its flanking sequences from its parent, Coker 176 (**Figure 3A**), accurately reflecting the *N*-fragment in Coker 176. By mapping deep sequencing reads from CB-1 and *N. glutinosa* to contig 094L, we identified that the sequences flanking the *N* gene in 0970A/Coker 176 are predominantly derived from *N. tabacum* on the left side and *N. glutinosa* on the right side, with the boundary between these two species’ sequences lying at approximately 2.73 Mb, slightly upstream of the *N* gene’s position at 2.94 Mb (**Figures 3B, C**). This indicates that the *N*-fragment from *N. glutinosa* retained in 0970A/Coker 176 spans approximately 3.74 Mb (**Figure 3C**), marking the site of genetic exchange during the breeding of Coker 176.

This study identified that contig 094L not only carries the *N* gene but also contains three *N*-like genes within its LSN region (a segment derived from *N. tabacum*) (**Supplementary Table S1**). Given that *R* genes can evolve into new *R* genes through tandem duplication (Panchy et al., 2016), it is plausible that these *N*-like genes may have originated from the *N* gene. To explore this possibility, we used Liftoff software (Shumate and Salzberg, 2021) to project the gene annotations from contig 094L to the corresponding region in the CB-1 genome and performed comparative sequence analysis. The results revealed that all three *N*-like genes are also present in CB-1 (**Supplementary Table S3**), suggesting that they are native to *N. tabacum* rather than derived from the *N* gene. Interestingly, sequence alignment of the *N* gene itself identified a homologous sequence at the corresponding locus in CB-1, although with relatively low sequence identity (**Supplementary Table S3**). The biological significance of this observation remains unclear.

A previous study by Lewis et al. (2005) identified two distinct types of *N. tabacum* germplasm carrying the *N* gene: one with the *N* gene located on chromosome H and the other on an unidentified chromosome. The pioneering variety Holmes Samsoun, which introduced the *N* gene from *N. glutinosa*, belongs to the first type, as does Coker 176 (Lewis et al., 2005). Based on the sequence composition of contig 094L, we can infer that the *N* gene is positioned 3.53 Mb from the end of a chromosome in *N. glutinosa*, and the *N*-fragment was integrated into chromosome H in Holmes Samsoun through a simple or reciprocal translocation. This suggests that the initial *N*-fragment introgressed from *N. glutinosa* was likely substantial. Due to limited genetic lineage data, it remains unclear whether the second type of germplasm also originated from Holmes Samsoun. Although chromosome H was initially identified using monosomic material (Clausen and Cameron, 1944), its correspondence in the sequenced tobacco genome was previously unknown. This study clarifies that chromosome H corresponds to chromosome Nt11.

Lewis et al. (2005) identified 168 N*. glutinosa*-specific AFLP markers linked to the *N* gene in Holmes Samsoun. Their analysis of 15 *N*-gene-carrying *N. tabacum* varieties (8 of the first type and 7 of the second) revealed that all varieties retained some of these markers, albeit in significantly reduced numbers (3 to 17 markers) compared to Holmes Samsoun. This reduction indicates that linkage drag from *N. glutinosa* was substantially diminished after multiple backcross generations. Notably, linkage drag was less pronounced in the first type compared to the second. Among the varieties, Coker 176 and another first-type variety, Burley 21, exhibited the least linkage drag, retaining only 3 specific markers. Our study further reveals that the *N*-fragment in 0970A/Coker 176 spans only 3.74 Mb, with linkage drag primarily persisting on the right side of the *N* gene and nearly eliminated (only ~210 kb remaining) on the left side (**Figure 2C**). This suggests that the *N*-fragment in Coker 176 is among the smallest achievable through traditional breeding methods based on phenotypic selection, making Coker 176 an optimal donor parent for TMV-resistance breeding in tobacco.

However, a recent study by Liu et al. (2023) reported that the *N*-fragment from Coker 176 can increase chlorophyll content and leaf thickness, leading to slower yellowing in the field, greater difficulty in curing, and reduced yield and output value. This indicates that the *N*-fragment from Coker 176 may still carry undesirable effects. Our study predicts that the *N*-fragment in 0970A/Coker 176 harbors up to 117 genes from *N. glutinosa*, including the *N* gene (**Supplementary Table S1**). These genes encompass a wide range of functions, suggesting that the *N*-fragment in Coker 176 has the potential for negative impacts. Notably, within the contig 094L, the gene density on the *N. glutinosa* sequence (117/2.74 = 31.3 genes/Mb) is approximately 70% higher than that on the *N. tabacum* sequence (50/2.73 = 18.3 genes/Mb) (**Supplementary Table S1**). Given the high gene density in the *N*-fragment, minimizing potential linkage drag is critical to mitigating its adverse effects.

In this study, we introgressed the *N* gene from 0970A into CB-1 through backcross breeding, successfully eliminating 92.8% (3.47/3.74 × 100%) of the *N*-fragment from 0970A/Coker 176. This resulted in TMV-resistant near-isogenic lines of CB-1 with only ~270 kb (7.2%) of the *N*-fragment remaining. The residual segment contains 17 predicted genes, apart from the *N* gene (**Supplementary Table S1**). Interestingly, approximately 70% of these genes belong to the auxin-responsive protein SAUR (small auxin-up RNA) gene family, which is implicated in cell elongation and may also play roles in processes such as leaf senescence and cell division (Stortenbeker and Bemer, 2019). Additionally, SAUR genes are regulated by plant hormones such as ABA and JA, in addition to auxin (Stortenbeker and Bemer, 2019). Whether these genes in the residual *N*-fragment negatively impact flue-cured tobacco production remains to be investigated. The near-isogenic lines of CB-1 developed in this study provide an excellent resource to address this question.

To our knowledge, the TMV-resistant near-isogenic lines of CB-1 developed in this study represent the tobacco materials with the least residual *N*-fragment. These lines not only facilitate the breeding of TMV-resistant CB-1 without the undesirable effects of linkage drag but also serve as ideal *N*-gene donors for TMV resistance breeding in tobacco. Furthermore, this study provides a successful example of using genomic techniques combined with marker-assisted breeding to precisely analyze and minimize introgressed genomic fragments from wild species into cultivated species through distant hybridization.

## 5 Conclusion

In this study, we successfully addressed the challenge of linkage drag associated with the *N* gene in cultivated tobacco by combining advanced genome sequencing and marker-assisted breeding strategies. Through comprehensive genomic analysis, we pinpointed the *N* gene’s location and identified a 3.74 Mb introgressed fragment from *N. glutinosa* in the resistant line 0970A. By screening a large BC_4_F_1_ population, we obtained a recombinant plant with a drastically reduced *N*-fragment (~270 kb), significantly minimizing potential linkage drag while retaining robust TMV resistance. Our findings underscore the effectiveness of genomic tools in accelerating precision breeding and highlight the potential for applying similar approaches to other crop improvement programs. The developed TMV-resistant lines and molecular markers provide valuable resources for future tobacco breeding, paving the way for more efficient utilization of wild germplasm in enhancing crop resilience.

## Data Availability

The datasets presented in this study can be found in online repositories. The names of the repository/repositories and accession number(s) can be found in the article/**Supplementary Material**.
