# Supplementary material for: Genomic sequencing combined with marker-assisted breeding effectively eliminates potential linkage drag of a target gene: a case study in tobacco
Source: Front Plant Sci. 2025 Sep 24;16:1666106. doi: 10.3389/fpls.2025.1666106 (PMC12504484; doi:10.3389/fpls.2025.1666106)
Supplement: Supplementary file 1 [file DataSheet1.zip › Supplementary table S2.docx]

**Supplementary Table S2.** Statistics of the number of SNPs of different genotypes and percentage of CB-1 alleles in each recombinant or non-recombinant line

| Genotype | Non-recombinant A | Recombinant B | Recombinant C | Recombinant D |
| --- | --- | --- | --- | --- |
| 0970A | 3,270 | 1,013 | 1,153 | 574 |
| Heterozygous | 10,042 | 3,913 | 1,694 | 4,488 |
| CB-1 | 112,669 | 145,057 | 153,178 | 125,845 |
| Total | 125,981 | 149,983 | 156,025 | 130,907 |
| % of CB-1 alleles | 93.42 | 98.02 | 98.72 | 97.85 |

**Note:** The next-generation sequencing reads of the CB-1 genome (with an average sequencing depth of approximately 45) were aligned to all contigs of 0970A. Homozygous SNPs with a sequencing depth of 10 or greater were selected to form the SNP marker set. The next-generation sequencing reads of each line were then aligned to all contigs of 0970A, and SNP markers with a sequencing depth of 8 or greater were selected. The number of SNP markers with 0970A, heterozygous (HET), and CB-1 genotypes in each line was counted, and the percentage of CB-1 alleles was calculated as: % of CB-1 alleles = (CB-1 + HET/2) / Total.
