# Supplementary material for: Genomic sequencing combined with marker-assisted breeding effectively eliminates potential linkage drag of a target gene: a case study in tobacco
Source: Front Plant Sci. 2025 Sep 24;16:1666106. doi: 10.3389/fpls.2025.1666106 (PMC12504484; doi:10.3389/fpls.2025.1666106)
Supplement: Supplementary file 1 [file DataSheet1.zip › Supplementary figure S1,S2.docx]

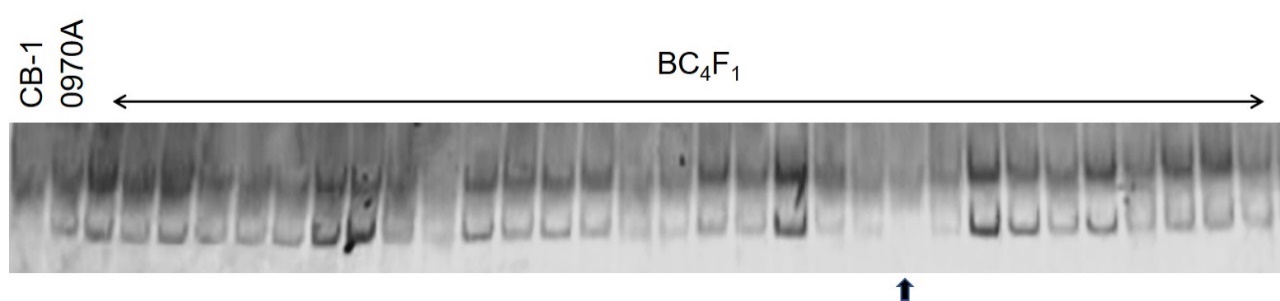


**Figure S1.** Genotyping results of molecular marker P371 in parents CB-1 and 0970A as well as some TMV-resistant BC_4_F_1_ plants. The arrow indicates a BC_4_F_1_ plant lacking the 0970A-specific amplification band, suggesting that recombination occurred between the P371 marker and the *N* gene in this plant.


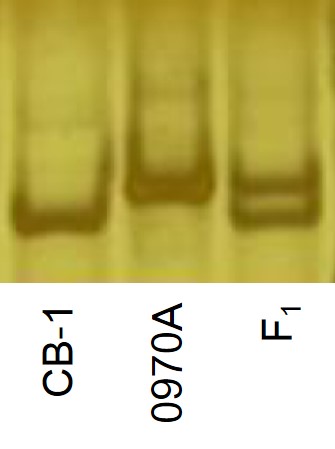


**Figure S2.** Genotyping results of molecular marker NR5 in CB-1, 0970A and their F_1_
